# Paediatric Emergency Triage, Assessment and Treatment (ETAT) – preparedness for implementation at primary care facilities in Malawi

**DOI:** 10.1080/16549716.2021.1989807

**Published:** 2021-11-13

**Authors:** Carina King, Albert Dube, Beatiwel Zadutsa, Lumbani Banda, Josephine Langton, Nicola Desmond, Norman Lufesi, Charles Makwenda, Helena Hildenwall

**Affiliations:** a Department of Global Public Health, Karolinska Institutet, Stockholm, Sweden; b Institute for Global Health, University College London, London, UK; c Malawi Epidemiology and Intervention Research Unit (Meiru), Lilongwe, Malawi; d Parent and Child Health Initiative, Lilongwe, Malawi; e Department of Paediatrics, College of Medicine, Blantyre, Malawi; f Department of International Public Health, Liverpool School of Tropical Medicine & Behaviour and Health Group, Malawi-Liverpool-Wellcome Trust Clinical Research Programme, Blantyren, Malawi; g Acute Respiratory Infections Unit, Ministry of Health, Lilongwe, Malawi; h Astrid Lindgren Children’s Hospital, Karolinska University Hospital, Stockholm, Sweden; i Department of Clinical Science, Intervention and Technology, Karolinska Institutet, Stockholm, Sweden

**Keywords:** Triage, primary care, emergency care, paediatrics, sub-Saharan Africa

## Abstract

**Background:**

The majority of deaths amongst children under 5 years are still due to preventable infectious causes. Emergency care has been identified as a key health system weakness, and referrals are often challenging.

**Objective:**

We aimed to establish how prepared frontline facilities in Malawi are to implement WHO Emergency Triage Assessment and Treatment (ETAT) guidelines, to support policy and planning decisions.

**Methods:**

We conducted a concurrent mixed-methods study, including facility audit; healthcare provider survey; focus group discussions (FGD) and semi-structured interviews with facility staff. The study was conducted in two districts in Malawi, Zomba and Mchinji, between January and May 2019. We included all frontline facilities, including dispensaries, primary health centres, rural and community hospitals. Quantitative data were described using proportions, means and linear regression. Qualitative data was analysed using a framework approach. Data were analysed separately and then triangulated into common themes.

**Results:**

Forty-seven facilities and 531 healthcare providers were included in the audit and survey; 6 FGDs and 5 interviews were completed. Four common themes emerged: (1) current emergency case management; (2) referral practices; (3) trained staff capacity; (4) opportunities and barriers for ETAT. Triage was conducted in most facilities with various methods described, and 53% reporting all staff are responsible. Referrals were common, but challenging due to issues in transportation. Twelve percent of survey respondents had ETAT training, with clinical officers (41%) reporting this more frequently than other cadres. Training was associated with increased knowledge, independent of cadre. The main barriers to ETAT implementation were the lack of resources, but opportunities to improve quality of care were reported.

**Conclusions:**

Malawian frontline facilities are already providing a level of emergency paediatric care, but issues in training, drug supplies and equipment were present. To effectively scale-up ETAT, policies need to include supply chain management, maintenance and strengthening referral communication.

## Background

The majority of deaths amongst children under 5 years are still due to preventable infectious causes, namely pneumonia, diarrhoea and malaria [1]. National and global initiatives to improve access to basic care through standardised programmes such as the World Health Organisation’s (WHO) Integrated Management of Childhood Illness (IMCI) and integrated Community Case Management (iCCM) [2,3] have contributed to reductions in paediatric mortality [4]. Both IMCI and iCCM were designed for primary care settings to promote access and early initiation of first-line treatment or referral of severe cases to inpatient facilities. However, the Commission on High Quality Health Systems 2018 report estimated 60% of global preventable deaths occur from poor quality care, rather than challenges with access [5].

The provision of emergency care has been highlighted as a weakness of healthcare systems in low- and middle-income countries (LMICs) [6]. There are two strategies for managing severely ill patients in primary care: either stabilise and refer or urgently refer. For severely sick children, both iCCM and IMCI recommendations are to provide pre-referral treatment (e.g. first dose of antibiotics/antimalarial) and urgently refer. However, referrals are commonly difficult, with financial constraints and transportation barriers [7–9]. As a consequence, children frequently arrive at hospitals at a very late stage of illness, limiting the ability to effectively treat the child [10]. A recent study from Malawi reported 33% of paediatric deaths in a tertiary facility occurred in the first 24 h of admission [11].

The WHO’s Emergency Triage Assessment and Treatment (ETAT) guidelines were designed as a practical approach to rapidly identify and then treat severely sick children [12]. There is a clear overlap between IMCI and ETAT assessments, with the intention that they are complimentary [13]. A subsequent adaptation, ETAT+, includes admission care [14]. There are examples of successful implementation in LMIC hospital settings and economic evaluation suggests cost-effectiveness at scale [15]. Therefore, to achieve Sustainable Development Goal 3.2 – the elimination of preventable childhood deaths by 2030, further implementation of quality paediatric emergency care is likely to be a key strategy.

In Malawi, ETAT has been implemented in tertiary and secondary hospitals [16], with discussions about integration with the IMCI programme and roll-out to primary care at the time of the study [17]. A pilot study in peri-urban primary care in Malawi suggested implementation is feasible and increased accuracy of referrals; however, this study used a digital tool with decision support [18,19]. Given primary care facilities are mostly staffed by lower cadres of healthcare workers and varying resources, assessing the current capacity to deliver ETAT in this context is needed. We therefore aimed to establish the availability of equipment, medications, infrastructure, personnel and current practice as measures of preparedness of frontline health facilities in Malawi to implement ETAT.

## Methods

We conducted a mixed-methods study using a concurrent triangulation design (QUAN + QUAL) [20], with data collected simultaneously from facility audits, healthcare provider surveys, focus group discussions (FGDs) and semi-structured interviews with facility staff. The study was conducted in two districts in Malawi, Mchinji (central region) and Zomba (southern region) between January and May 2019, in facilities not implementing ETAT at the time of study.

### Setting

Mchinji has a population of approximately 600,000 and is served by a district hospital, four rural hospitals, and 14 primary healthcare facilities. The Zomba district has a population of 750,000, with a tertiary hospital, 4 rural hospitals, and 25 primary healthcare facilities [21]. Healthcare is provided free of charge in government facilities, and small user fees are charged by facilities run by the Christian Health Association of Malawi (CHAM). The under-five mortality rates reported in 2015/16 were 123 and 54 per 1,000 live births in Mchinji and Zomba, respectively. Overall, the Southern region of Malawi has a higher wealth status than other regions [22].

### Quantitative data collection

All health centres, dispensaries and rural hospitals in Zomba and Mchinji, which were operational and serving children under 5 years, were included in the audit, based on lists provided by District Health Management offices. A team of three study staff, including one clinical officer (BZ), visited each facility at a pre-arranged time to conduct the audit and surveys. Audit data was collected through visual inspection and responses to closed questions by the facility in-charge and other relevant staff members (e.g. pharmacy assistants). The audit included infrastructure, drug stocks, equipment availability, staffing and referral capacity and practice.

A convenient sample of facility staff, who were present at the time of data collection and interact with children, were recruited. Staff groups included attendants and support staff, health surveillance assistants (HSAs), nurses and midwives, medical assistants and clinical officers. The survey covered work experience, training, knowledge of ETAT, and current clinical practice including referral. ETAT knowledge consisted of 16 questions, with a mix of multiple choice and free-text; these questions were marked out of a total score of 22, with multiple component questions given a point for each correct item (***Web-Appendix 1***). The structured questionnaire was translated into Chichewa and self-completed by respondents on an Android tablet using CommCare, with the support of study staff.

### Qualitative data collection

We conducted six FGDs with healthcare providers to explore their understanding of ETAT, their perceived barriers to implementation and potential opportunities, and what their current approach to dealing with emergencies involves. We targeted three groups in each district: 1. HSAs and attendants at health centres; 2. medical assistants and nurses at health centres; 3. clinical officers, medical assistants or doctors at rural hospitals. Between 5 and 10 potential participants were invited per group. Semi-structured interviews were conducted with facility in-charges to explore patient management and how the implementation of ETAT would change current clinic practices in more depth. We purposefully selected eight facility in-charges for the interviews based on their facility being urban/peri-urban or rural, and small or large in terms of the patient load; we completed five interviews due to time constraints.

Participants were invited by phone by a member of study staff, with permission from the District Health Management Team and senior HSAs (who supervise the HSAs). Both interviews and FGDs followed topic guides, developed and reviewed by multiple members of the study team (***Web-appendix 2 and 3***). They were conducted at facilities by a male Malawian researcher (AD) with extensive experience of qualitative research, who resides outside of the study area with no prior relationship to participants. He was supported by a study clinical officer with subject specific knowledge (BZ). Discussions were planned in Chichewa, but preference of language was left up to participants. Interviews and discussions were audio-recorded and transcribed and translated (where necessary) into English. Group discussions took 60–90 minutes, and interviews 20–30 minutes. Participants were given refreshments, and any incurred transport costs were reimbursed.

### Analysis

The quantitative data were described, using counts, proportions and medians, and presented according to facility type, district and healthcare provider type where appropriate. A linear regression analysis was done to explore associations between healthcare worker ETAT knowledge scores and training experience, district or type of facility and word cadre; *p*-values <0.05 were considered statistically significant. All analyses were done using Stata SE14.

FGD and interview data were analysed separately using content analysis with a pragmatic framework approach [23]. Transcripts were coded inductively, as we did not have a pre-defined framework. FGDs were coded by HH, and interview data by CK. A sub-set of each were double coded, codes updated and then applied. The themes from FGDs and interviews were then derived through discussion and shared with AD to ensure discussions had been accurately understood. The final interpretation was shared with the wider study team for input.

Qualitative and quantitative data were analysed separately, then triangulated occurred at the stage of presenting and interpreting the results, with the purpose of converging and corroborating findings [20]. This was done by CK, HH and AD reviewing codes, themes, figures and tables and then discussing where there was an overlap to create common themes. Results are presented together under these themes.

### Ethics

Consent for facility audits was granted by the District Health Management Team in each district. Individual verbal informed consent was sought for healthcare provider surveys and was recorded in the data collection form in the tablet. Written consent was gained for qualitative discussions and interviews, following an explanation of the purpose. The study was reviewed and approved by the College of Medicine Research Ethics Committee in Malawi (reference: P.11/18/2538).

## Results

**Figure 1.**
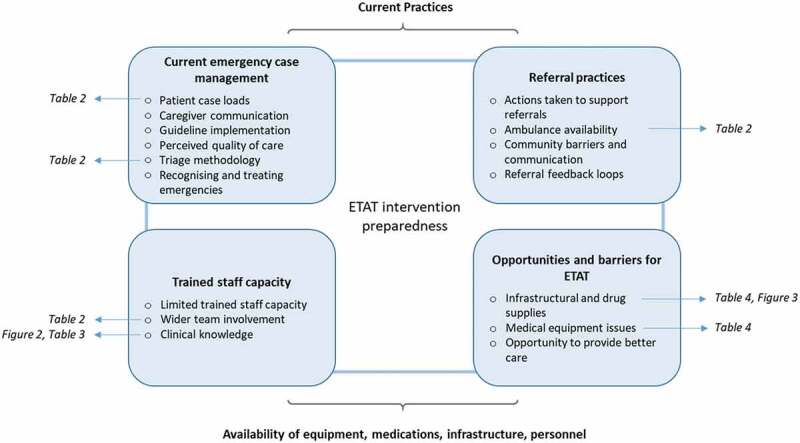
Schematic of themes and sub-themes

In Zomba, 31 of 40 facilities were included; one not currently providing services, a central and three community hospitals and four facilities not providing routine care for children were excluded. In Mchinji 16 of 19 facilities were included; two health centres not currently providing services and the district hospital were excluded. Overall six FGDs and five semi-structured interviews were conducted, and 531 healthcare providers completed the questionnaire (Table 1), of which 397 (75%) were clinical staff. Amongst the non-clinical staff, 47% reported providing emergency care to a child. We present quantitative and qualitative data together under the following themes (Figure 1): current emergency case management, referral practices, trained staff capacity and opportunities and barriers for ETAT.

**Table 1.** Summary of study facilities and participants

		Zomba district		Mchinji district	
		Facilities (N = 31)	Providers (N = 338)	Facilities (N = 16)	Providers (N = 193)
Facility type	Dispensary Health centre Rural hospital	3 24 4	26 (8%) 286 (85%) 26 (8%)	2 10 4	14 (7%) 92 (48%) 87 (45%)
In-charge/provider type	Clinical officer Medical assistant Nurse/Midwife HSA Attendant	6 16 9 - -	11 (3%) 32 (9%) 73 (22%) 121 (36%) 101 (30%)	1 11 4 - -	6 (3%) 15 (8%) 32 (17%) 69 (36%) 71 (37%)
Focus group discussions		3		3	
Interviews		3		2

### Current emergency case management

Current emergency case management includes the following sub-themes: case load, recognising and treating emergencies, perceived quality of care, triage methodology, and guideline implementation.

Case load: There was considerable variability in the number of absolute and severe cases reported at different facilities (Table 2). In the qualitative discussions, some variability was attributed to seasonality, but overall caseloads were considered high and severe cases to be common:

*As a clinician, the standards say I need to see about 20 patients in a day, but currently I see about 600 [patients] or more in the day*. (Zomba, Interview 3)

**Table 2.** Current triage and referral practices

		Dispensary	Health centre	Rural hospital
Triage conducted	Yes	27 (68%)	279 (74%)	84 (74%)
	No	10 (25%)	66 (17%)	16 (14%)
	Unsure	3 (8%)	33 (9%)	13 (12%)
Staff conducting triage (*n* = 390)	All staff	16 (59%)	163 (58%)	53 (63%)
	Clinical + non-clinical staff	3 (11%)	49 (18%)	12 (14%)
	Clinical staff only	4 (15%)	34 (12%)	7 (8%)
	Non-clinical staff only	4 (15%)	31 (11%)	12 (14%)
Triage approach (*n* = 390)	ETAT	2 (7%)	60 (22%)	18 (21%)
	IMCI danger sign	10 (37%)	80 (29%)	17 (20%)
	Child’s appearance	13 (48%)	126 (45%)	46 (55%)
	Caregiver indication	2 (7%)	13 (5%)	3 (4%)
Ever called an ambulance	Yes	9 (23%)	115 (30%)	42 (37%)
	No	31 (78%)	263 (70%)	71 (63%)
Three main reasons for not calling an ambulance (*n* = 365)		Quicker using own transport	No emergencies	Have their own transport
		Ambulances don’t go there	Quicker using own transport	No emergencies
		Roads too bad	Ambulances don’t go there	No fuel
Three main reason for calling an ambulance (*n* = 166)		Malaria (78%)	Pneumonia (38%)	Pneumonia (29%)
		Pneumonia (11%)	Malaria (31%)	Anaemia (21%)
		Anaemia (11%)	Anaemia (17%)	Accidents (12%)
Hours for ambulance to arrive (median; IQR)		2 (1.5–3)	3 (2–6)	2 (0.5–4)
Children seen per week (median; IQR)		38 (13–195)	20 (9–100)	25 (10–100)
Severe children seen per week (median; IQR)		5 (2–20)	5 (2–10)	7 (4–20)
Child referrals per week (median; IQR)		2 (1–7)	2 (1–5)	2 (1–3)

Recognising and treating emergencies: Common paediatric emergency presentations were described as high fevers, difficulty breathing, fainting and convulsions, dehydration, hypoglycaemia, severe malnutrition and accidents. Healthcare workers (HCWs) reported treating many of these at both health centres and rural hospitals, inserting IV lines for fluid and dextrose, resuscitation and keeping patients for observation and stabilisation.

Perceived quality of care: Challenges in being able to provide quality care due to lack of equipment and supplies were apparent, and HCWs described cases where they were unable to provide the care they wanted. There was general consensus that they tried their best to implement alternative solutions:

*I knew that I needed to provide fluids to the baby but failed. A lack of resources made me fail in providing the necessary management for the baby. So I transferred the baby to Zomba central hospital with oral fluids. I managed to provide oral fluids […] I just made the solution and gave it to the mother to give to the child and [advised her to] breastfeed on her way to the hospital*. (Zomba, FGD 3)

Triage methodology: 73% of survey respondents reported triaging within their clinic, with similar rates across facility types (Table 2). There were differences in the approach to triage used, with more using ETAT at health centres and rural hospitals than dispensaries, and a higher number of providers in Zomba reporting using ETAT than in Mchinji (25% versus 14%). However, from the qualitative data, it was not clear whether those reporting implementing ETAT were familiar with this in practice:

*I know the meaning but don’t understand what it does*. (Zomba, Interview 1)

Various approaches to triaging were described, including one clinic using colour coding, looking for specific signs such as convulsions and prioritising children over adults. However, it was noted that triage can be problematic if other patients think that their issue is more important or do not understand why others are seen before them.

*Ideally before splitting the groups [patients/caregivers] are told to start sitting on the third bench, avoiding the red bench which has been reserved for those that have been triaged as very sick and the yellow bench is for the priority*. (Zomba, Interview 3)

Guideline implementation: All HCWs discussed the use of guidelines to support case management, and using IMCI for severe cases. However, some participants acknowledged that guidelines were not always followed and could cause problems for HCWs if they increased the complexity of their job. Discussions explored how guidelines are introduced at the facility, and this was generally done through facility meetings, and key to acceptance was participation in adoption.

*We throw it to the participants, because we are at least 37 [staff] here, so it’s a good ideal. So we say, there is this protocol, we need to implement it, how can we go about it, do we think we can have problems. […] After tabling them, this message is just spread to the general staff - so this is what we do*. (Mchinji, Interview 2)

### Referral practices

Referral practices include the sub-themes: ambulance availability, community barriers and communication, actions taken to support referrals, and referral feedback loops. Of the survey respondents, 11% had referred a child on the day of the survey and a median of 2 (range: 0–50) children per week.

Ambulance availability: The three most common reasons given for referral were pneumonia (26%), malaria (26%) and anaemia (17%); this broadly reflects the reasons given for calling an ambulance, with the exception of rural hospitals (Table 2). The challenges in ambulance availability were reflected in the qualitative discussions, with all participants raising this issue. One group of participants discussed that the ambulance is reserved for maternity cases, limiting their ability to refer child cases:

*I remember even the [District Health Office] has accepted this, because he communicated that the people that brought the ambulance did so for the maternity cases, so it is like there is nothing that can be done about it*. (Zomba, FGD 3)

Community barriers and communication: Perceived community barriers to successful referrals focused predominantly on transport, and funding this. However, one participant noted that the seriousness of the child’s condition was not always appreciated by guardians, leading to non-adherence. One participant also described how caregivers might view them negatively when they refer, perceiving them as unable to provide adequate care.

Actions to support referrals: Examples of both HCW and community actions to support referrals included finding other means of transport, loaning funds and counselling them on the need to attend.

*Yes I remember it one time … looking at the condition of the child and the economic status of the mother I decided to give them 2000 kwacha and I asked the nurse to assist them to get a taxi to take them to Zomba*. (Zomba, Interview 2)

Feedback loops: From the audit, 18 (38%) facilities used referral slips, and 37 (79%) reported that they do not actively follow-up referred cases. From the qualitative discussions, there was a clear desire for feedback about referred cases, to know the outcome for the child but also for professional development. Many HCWs noted that they do not know what happens to children after they refer them. However, it was not consistent whether feedback should come directly from the hospital or through the District Health Office.

*The problem is we do not share feedback, otherwise we would be learning a lot from this feedback* (Zomba, FGD 3)

### Trained staff capacity

This theme has three sub-themes: limited trained staff capacity, clinical knowledge and wider team involvement in triage.

Limited trained staff capacity: Of the 531 survey respondents, 12% reported ETAT training. This was most common in clinical officers (41%) and nurses (31%), with very few trained HSAs and attendants (3%). Providers in Zomba were significantly more likely to report training than those in Mchinji (15% versus 6%, *p*-value = 0.004), despite similar proportions of HCW cadres. Training was predominately received during qualification (57%), a mean of 5.5 years prior to the survey (range: 1–11 years), and only four reported a subsequent refresher. The lack of trained staff in emergency case management was echoed in the qualitative discussions. HCWs reported there being enough staff to support ETAT implementation, but not enough had training. One reason being staff turnover, if the whole team is not trained, then facilities lose skills when individuals leave, although the converse was also seen as a benefit.

*Swapping really affects delivery because there are prominent members or health workers, which you know this one went for a certain training, maybe for malaria or EPI or STI, that one goes out. Or it may be [the person who] might come who is not yet trained […] it becomes a problem*. (Mchinji, Interview 2)

Clinical knowledge: Out of maximum 22 points, the mean knowledge score was 4.9 (range: 0–19; Figure 2), with clinical officers scoring the highest (13.6; range: 8–18). Those with ETAT training scored significantly higher than those without (11.4 versus 4.1, *p*-value <0.001). Notably, only 10% of the respondents scored 50% or more. The question with the highest correct responses was defining stridor (62%). The lowest was a fluid treatment for severe dehydration (2%), with no difference by training – ***Web-Appendix 4***. Amongst those who had received training, there was a negative association between time since training and knowledge (coefficient: −0.80; 95% CI: −1.26, −0.34). HCW cadre remained significantly associated with knowledge, independent of training (Table 3).

**Table 3.** Linear regression analysis of ETAT knowledge

Variable		Coefficient	95% Confidence interval		p-value
Training	No	*Ref*			
	Yes	2.71	1.99	3.44	<0.001
District	Mchinji	*Ref*			
	Zomba	0.01	−0.48	0.50	0.969
Facility type	Dispensary	*Ref*			
	Health centre	0.17	−0.63	0.97	0.674
	Rural hospital	0.74	−0.18	1.65	0.115
Provider type	Clinical officer	*Ref*			
	Medical assistant	−1.94	−3.31	−0.56	0.006
	Nurse/midwife	−3.26	−4.52	−1.99	<0.001
	HSA	−10.47	−11.74	−9.20	<0.001
	Hospital attendant	−9.97	−11.24	−8.71	<0.001

**Figure 2.**
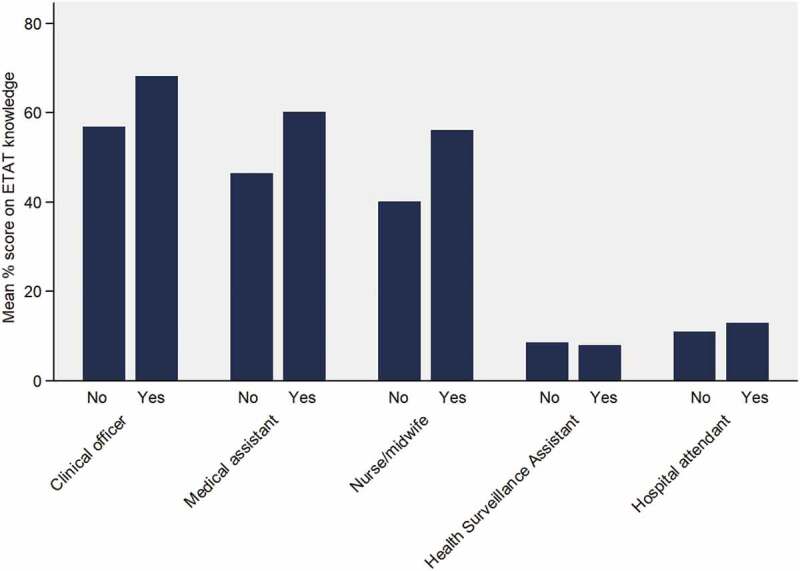
Proportion of maximum ETAT knowledge score for different healthcare provider types, according to ETAT training

Wider team involvement: From the survey, 53% answered that all clinic staff were involved in triage. This was reflected in discussions, with triage seen as everyone’s responsibility to ensure emergency cases were prioritised. It was requested that all staff, including cleaners and guards, be trained. This would allow them to support when a clinician is not available (e.g. outside clinic opening hours) or when more hands are needed to treat an emergency case. There was general consensus that all cadres of facility staff and caregivers recognise emergency cases:

*Sometimes the guardians themselves, they just see that the child of their friend is very sick, they let their friend with a very sick child be attended first, they see dangerous signs on that child*. (Mchinji, Interview 2)

### Opportunities and barriers to ETAT delivery

Sub-themes included: medical equipment issues, infrastructural and drug supplies, and the opportunity to provide better care.

Medical equipment issues: Availability of functional ETAT equipment was variable, with a resuscitation bag and mask and glucometer found in ≥50% of facilities, while haemoglobin testing equipment was more limited (Table 4). Several items of essential ETAT equipment require a power source, either for charging or as a continuous supply; all facilities had access to power, except one dispensary. Those with power were either connected to the mains system (*n* = 35, 74%) or had solar power (*n* = 11, 23%). Amongst those with power, 34 (74%) reported 24 hours of power the day before.

**Table 4.** Presence of essential ETAT equipment and drugs

	Zomba (N = 31)	Mchinji (N = 16)
**Drugs and medications (% in stock, median number and range)**		
Dextrose 50%	22 (71%; 25, 1–140)	15 (94%; 254, 8–3091)
Adrenaline	16 (52%; 91, 7–1000)	13 (81%; 20, 9–90)
Atropine	5 (16%; 52, 10–172)	14 (88%; 33, 10–80)
Diazepam	29 (90%; 68, 7–160)	13 (81%; 64, 10–122)
Intravenous ceftriaxzone	9 (29%; 21, 4–70)	8 (50%; 91, 4–360)
Intravenous benzylpenicillin	31 (100%; 111, 6–400)	15 (94%; 152, 2–500)
Intravenous gentamicin	31 (100%; 1400, 72–5500)	16 (100%; 2647, 200–4929)
Intravenous artesunate	29 (94%; 229, 12–852)	16 (100%; 1174, 10–7303)
**Functional equipment (n, %)**		
Oxygen	9 (29%)	5 (31%)
Pulse oximeter	4 (13%)	7 (44%)
Resuscitation bag and mask	22 (71%)	8 (50%)
Glucometer	17 (55%)	13 (81%)
Glucose test strips	9 (29%)	10 (63%)
Haemoglobin testing	16 (52%)	3 (19%)
Haemoglobin cuvettes	7 (23%)	3 (19%)

The qualitative discussions reflected barriers in medical equipment and that even when equipment was present and functional, this did not necessarily translate into being able to provide optimal care. Specific examples included having a glucometer but no test strips, needing to start a blood transfusion but no way to test blood type, having oxygen but no splitters to enable treatment of multiple patients, and an old pulse oximeter that no longer switches between paediatric and adult modes.

*In our case we have a glucometer which we can use, but when we went to the [District Health Office] to order the strips, we were told they don’t match. As a result, it is just as good to say we don’t have the glucometer*. (Zomba, FGD 3)

**Figure 3.**
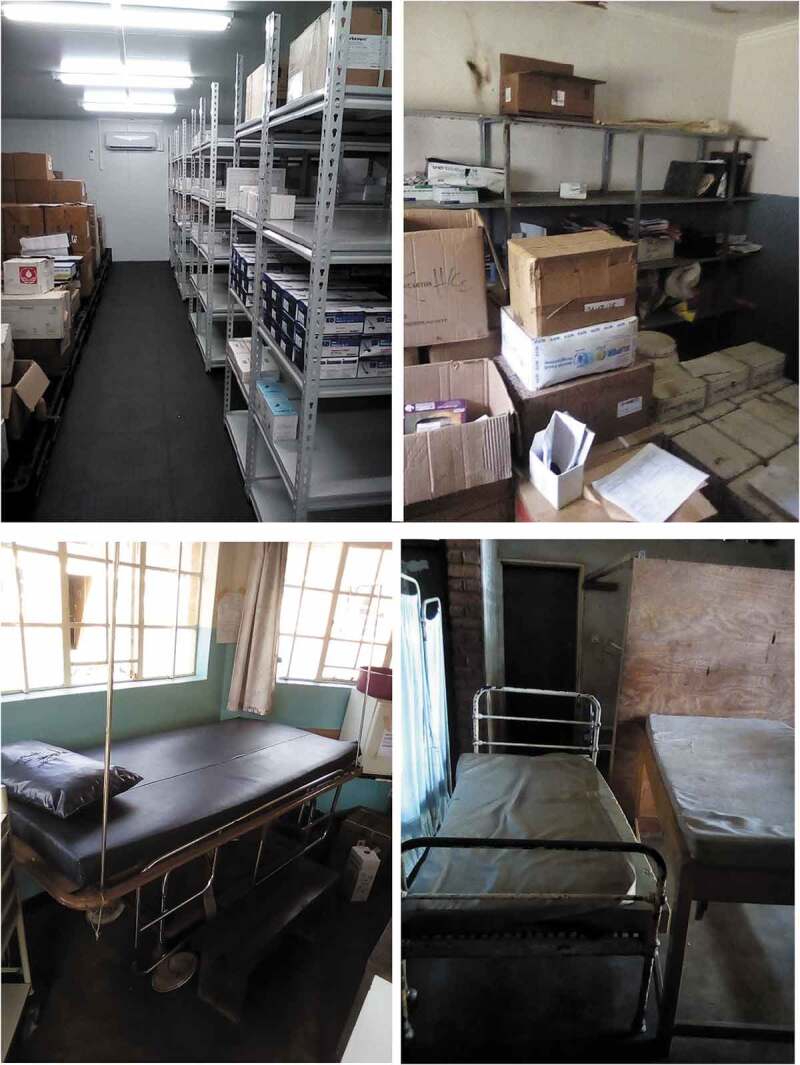
Examples of drug stores and triage rooms. *a) Newly built drug store with temperature control in Mchinji District; b) Drug store without temperature control in Zomba District; c) short term stabilisation bed in Mchinji District; d) side-room for triage and stabilisation in Zomba district.*

Infrastructural and drug supplies: All facilities reported dedicated drug storage facilities (Figure 3); 72% (*n* = 34) had temperature control function, and this was less common in dispensaries (*n* = 4/7) compared to rural and CHAM hospitals (*n* = 6/8). Seventy percent (*n* = 33) of facilities reported a stock-out of essential medicines within the month prior to audit and additional three clinics in the prior three months. The antibiotic intravenous ceftriaxone is the least commonly available (Table 4). Both lack of drugs and insufficient infrastructure were raised in the FGDs from Mchinji and Zomba.

Opportunity to provide better care: Two areas for better care were raised – quick treatment and a desire to provide good-quality care. Across discussions, HCWs were supportive of ETAT introduction, motivated by the ability to treat children quickly and therefore improve outcomes. It was also seen that this would positively affect patient flow and workloads, both at frontline facilities and at hospitals through reducing referrals. One respondent commented that reducing referrals would result in cost-savings to caregivers.

*They are quite a number of benefits but the first one that I see is that we want to save lives […]. Secondly it lessens panic on the part of the practitioner because if he has already isolated those children with high fever there will be no cases to panic with*. (Zomba, Interview 3)

## Discussion

We explored current emergency case management and triaging practice in frontline health facilities in two districts in Malawi. Overall, most facilities and HCWs were familiar with triage, and emergency paediatric cases were common. Notable barriers to delivering quality emergency care were seen, in particular transport for referrals, trained staff time and adequate supplies. Clear opportunities were also presented, with multiple local adaptations to overcome these barriers, the desire to offer more comprehensive emergency services and wider staff participation in triage.

Referrals have long been recognised as a difficult process and a weak link in health systems in Malawi and other low-income countries. Many of the issues with transport, ambulance availability and costs are in line with previous studies [17,24], as was the use of personal and community resources to help children get to hospital [25]. While a key request from healthcare workers was referral feedback loops, we note that frontline facilities also reported not actively attempting follow-ups. This points to a lack of systems and procedures to support communication. A paediatric critical care electronic medical record system was recently developed at a tertiary hospital in Malawi, and while it includes fields for pre-referral care, feedback loops were not incorporated [26]. Therefore, efforts are needed to overcome unilateral information flow, such as mHealth facilitated systems, referral communication training, and comprehensive linked medical records in primary care.

Given the difficulty of urgent referral, it was apparent that many HCWs attempt to stabilise patients within frontline facilities. Several adaptations to lacking resources were discussed, and reflects similar challenges in treating emergency cases reported from a hospital setting in Malawi [27]. Indeed, the lack of equipment and drugs to treat emergency cases has been reported in referral centres, which should be implementing ETAT [28]. Therefore, a key policy consideration for scaling ETAT to frontline facilities, is whether this will divert needed resources away from secondary and tertiary levels referral centres.

Overall facilities in Mchinji district were better stocked with both equipment and drugs, while more HCWs in Zomba reported implementing ETAT. This may reflect local priorities, external programmes or research investments. One example being pulse oximetry implementation in Mchinji as part of a pneumococcal conjugate vaccine evaluation in 2012, which likely explains the higher numbers of pulse oximeters found in the district (44% vs. 13%) [29]. Alternatively, with more training, drugs may be more readily administered, and therefore result in more frequent stock-outs. This would be compounded when access to and referrals from facilities proves particularly challenging; at the time of data collection, Zomba was experiencing heavy rains and localised flooding. Given that effective supply chain management and aligned budgets are a key component of quality service delivery, this warrants further exploration before a policy scale-up.

While ETAT knowledge was high in those who had received recent training, and who would regularly attend paediatric emergency cases, there were gaps. A notable example was around fluid management. While those with training responded better on shock treatment than for dehydration, still only one-third were correct. Challenges in dehydration likely reflect differentiated recommendations depending on whether the child is presenting with dehydration or impaired circulation due to infection, requiring a higher degree of assessment and diagnostic consideration. Along with training, it will be important to ensure ongoing clinical mentorship and skills building if ETAT is rolled-out to frontline facilities. Our findings relate to Malawi specifically, but similar challenges in the management of severely sick children are known from other low-income-countries, including attempts to roll out ETAT [30,31]. Improved patient management at primary care is associated with quality referrals, reduced admission days and complications and overall better patient outcomes [32]. As Malawi pioneered ETAT within hospitals and is now considering extending the ETAT programme to primary care facilities, it is an important case study.

We had key limitations in our study, firstly that we did not include all staff working at facilities. The convenience sample represents the healthcare providers present at the facility, therefore we would not have included those who focus on outreach services. We assumed that the main factor for not being present was rotation schedules rather than the characteristics of the provider. Second, the two districts assessed may not represent the rest of the country, with no data from the Northern Region included. This study would be strengthened with more contextual information on district-level budgets, resource allocation and management structures. We recommend that these data are collected from other regions prior to policy finalisation. Third, while we checked if equipment was functional, we did not assess whether healthcare providers used it correctly or if oxygen concentrators were delivering safe medical oxygen. Finally, we did not complete all the planned semi-structured interviews due to logistical time constraints, meaning we had a relatively small sample. While recruitment for interviews and FGDs was a pragmatic decision, data saturation was apparent in several themes.

## Conclusion

It is apparent that frontline facilities in Malawi already provide a level of emergency paediatric care, although quality of this care is hampered by trained staff and resource capacity. There was a clear desire to be able to manage these cases more effectively, and the opportunity to involve all cadres of healthcare workers in the triage process. To effectively scale-up ETAT to frontline facilities, strategies are needed to ensure that quality of care at referral facilities is not compromised and that those in need of urgent referrals are not inappropriately delayed. Key areas of focus will need to include supply chain management for an expanded package of drugs, equipment maintenance and uninterrupted power to support investments in oxygen and strengthening referral communication systems.

## Paper context

Given the challenges commonly faced in referrals from frontline to secondary care, strengthening emergency care in primary care through initiatives such as ETAT may be effective in reducing childhood mortality. We found that in Malawi, frontline facilities are already providing a level of emergency paediatric care. However, this is hampered by a lack of trained staff and resource capacity, and there was a clear desire to be able to manage these cases more effectively.

## References

[1] Liu L, Oza S, Hogan D, et al. Global, regional, and national causes of under-5 mortality in 2000–15: an updated systematic analysis with implications for the Sustainable Development Goals. Lancet. 2016;388:3027–11. DOI:10.1016/S0140-6736(16)31593-8. 27839855 PMC5161777

[2] World Health Organisation. Integrated management of childhood illness chart booklet. 2014.

[3] World Health Organisation. Joint statement on integrated community case management. World Health Organization and UNICEF: Geneva, Switzerland. 2012.

[4] Bhutta ZA, Das JK, Walker N, et al. Interventions to address deaths from childhood pneumonia and diarrhoea equitably: what works and at what cost? Lancet. 2013;381:1417–1429. DOI:10.1016/S0140-6736(13)60648-0. 23582723

[5] Kruk ME, Gage AD, Arsenault C, et al. High-quality health systems in the Sustainable Development Goals era: time for a revolution. Lancet Glob Health. 2018;6:e1196–e252. 30196093 10.1016/S2214-109X(18)30386-3 PMC7734391

[6] Nolan T, Angos P, Cunha AJLA, et al. Quality of hospital care for seriously ill children in less-developed countries. Lancet. 2001;357:106–110. DOI:10.1016/S0140-6736(00)03542-X. 11197397

[7] Peterson S, Nsungwa-Sabiiti J, Were W, et al. Coping with paediatric referral–Ugandan parents’ experience. Lancet. 2004;363:1955–1956. PubMed PMID: 15194257 15194257 10.1016/S0140-6736(04)16411-8

[8] Manongi R, Mtei F, Mtove G, et al. Inpatient child mortality by travel time to hospital in a rural area of Tanzania. Trop Med Int Health. 2014;19:555–562. 24661618 10.1111/tmi.12294 PMC4269975

[9] Po O, Maina J, PN T, et al. Access to emergency hospital care provided by the public sector in sub-Saharan Africa in 2015: a geocoded inventory and spatial analysis. Lancet Glob Health. 2018;6:e342–e50. DOI:10.1016/S2214-109X(17)30488-6. 29396220 PMC5809715

[10] Molyneux E. Paediatric emergency care in developing countries. Lancet. 2001;357:86–87. Epub 2001/ 02/24. PubMed PMID: 11197442. DOI:10.1016/S0140-6736(00)03536-4 11197442

[11] Ngwalangwa F, Phiri C, and Dube Q, et al., Risk factors for mortality in severely ill children admitted to a tertiary referral hospital in Malawi. Am J Trop Med Health. 2019;10(3):670-675. . 10.4269/ajtmh.19-0127 PMC6726928 31287044

[12] World Health Organisation. Emergency Triage Assessment and Treatment (ETAT) course. World Health Organisation. Geneva, Switzerland. 2005.

[13] Gove S, Tamburlini G, Molyneux E, et al. Development and technical basis of simplified guidelines for emergency triage assessment and treatment in developing countries. WHO Integrated Management of Childhood Illness (IMCI) Referral Care Project. Arch Dis Child. 1999;81:473–477. PubMed PMID: 10569960; PubMed Central PMCID: PMC1718149. 10569960 10.1136/adc.81.6.473 PMC1718149

[14] Irimu G, Wamae A, Wasunna A, et al. Developing and introducing evidence based clinical practice guidelines for serious illness in Kenya. Arch Dis Child. 2008;93:799–804. DOI:10.1136/adc.2007.126508. 18719161 PMC2654066

[15] Barasa EW, Ayieko P, Cleary S, et al. A multifaceted intervention to improve the quality of care of children in district hospitals in Kenya: a cost-effectiveness analysis. PLoS Med. 2012;9:e1001238–e. Epub 06/12. PubMed PMID: 22719233. DOI:10.1371/journal.pmed.1001238. 22719233 PMC3373608

[16] Robison J, Ahmed Z, Durand C, et al. Implementation of ETAT (Emergency Triage Assessment And Treatment) in a central hospital in Malawi. Arch Dis Child. 2011;96:A74–A5. DOI:10.1136/adc.2011.212563.174.

[17] Robertson SK, Manson K, Fioratou E. IMCI and ETAT integration at a primary healthcare facility in Malawi: a human factors approach. BMC Health Serv Res. 2018;18:1014. DOI:10.1186/s12913-018-3803-5. 30594185 PMC6310991

[18] O’Byrne T, Nyirenda D, Perrin R, et al. Improving recognition of severe illness and patient pathways in primary health services using mHealth technology in urban Blantyre, Malawi. J Mob Technol Med. 2013; 2: 2–3.

[19] Mtisunge Joshua G, Marc YRH, Thomasena OB, et al. Clinical diagnosis in paediatric patients at urban primary health care facilities in southern Malawi: a longitudinal observational study. BMC Health Serv Res. 2020. DOI:10.21203/rs.3.rs-62403/v1. PMC7885577 33588848

[20] Schoonenboom J, Johnson RB. How to construct a mixed methods research design. Kolner Zeitschrift fur Soziologie und Sozialpsychologie. 2017;69: 107–131. Epub 07/05. PubMed PMID: 28989188. DOI:10.1007/s11577-017-0454-1. 28989188 PMC5602001

[21] National Statistical Office/Malawi. Malawi population & housing census - preliminary report. Zomba (Malawi): National Statistical Office; 2018.

[22] National Statistical OM, Icf. Malawi demographic and health survey 2015-16. Zomba (Malawi): National Statistical Office and ICF; 2017.

[23] Gale NK, Heath G, Cameron E, et al. Using the framework method for the analysis of qualitative data in multi-disciplinary health research. BMC Med Res Methodol. 2013;13:117. DOI:10.1186/1471-2288-13-117. 24047204 PMC3848812

[24] King C, Banda M, Bar-Zeev N, et al. Care-seeking patterns amongst suspected paediatric pneumonia deaths in rural Malawi [version 1; peer review: awaiting peer review]. Gates Open Res. 2020;4. DOI:10.12688/gatesopenres.13208.1. PMC7835598 33537557

[25] Sips I, Haeri Mazanderani A, Schneider H, et al. Community Care Workers, Poor Referral Networks and Consumption of Personal Resources in Rural South Africa. PLoS One. 2014;9:e95324. DOI:10.1371/journal.pone.0095324. 24781696 PMC4004532

[26] Ciccone EJ, Tilly AE, Chiume M, et al. Lessons learned from the development and implementation of an electronic paediatric emergency and acute care database in Lilongwe, Malawi. BMJ Glob Health. 2020;5:e002410. DOI:10.1136/bmjgh-2020-002410. PMC7368472 32675067

[27] Lindsjö C, Chirambo CM, Langton J, et al. ‘We just dilute sugar and give’ health workers’ reports of management of paediatric hypoglycaemia in a referral hospital in Malawi. Glob Health Action. 2018;11:1491670. DOI:10.1080/16549716.2018.1491670. 30014776 PMC6052417

[28] EW J, Lindsjö C, Dj W, et al. Accessibility of basic paediatric emergency care in Malawi: analysis of a national facility census. BMC Public Health. 2020;20:992. DOI:10.1186/s12889-020-09043-3. 32580762 PMC7315502

[29] McCollum ED, King C, Deula R, et al. Pulse oximetry for children with pneumonia treated as outpatients in rural Malawi. Bull World Health Organ. 2016;94:893–902. PubMed PMID: 27994282; PubMed Central PMCID: PMC5153930. DOI:10.2471/BLT.16.173401. 27994282 PMC5153930

[30] Hategeka C, Mwai L, Tuyisenge L. Implementing the Emergency Triage, Assessment and Treatment plus admission care (ETAT+) clinical practice guidelines to improve quality of hospital care in Rwandan district hospitals: healthcare workers’ perspectives on relevance and challenges. BMC Health Serv Res. 2017;17:256. PubMed PMID: 28388951; PubMed Central PMCID: PMC5385061. DOI:10.1186/s12913-017-2193-4. 28388951 PMC5385061

[31] Kapoor R, Sandoval MA, Avendano L, et al. Regional scale-up of an Emergency Triage Assessment and Treatment (ETAT) training programme from a referral hospital to primary care health centres in Guatemala. Emer Med J. 2016;33:611–617. PubMed PMID: WOS: 000383270600004. DOI:10.1136/emermed-2015-205057. 27207345

[32] World Health O. Primary health care and health emergencies. Geneva:World Health Organization;2018. Report No.: Contract No.: WHO/HIS/SDS/2018.51.

